# A rare cause of vertebral osteomyelitis: the first case report of rat-bite fever in Portugal

**DOI:** 10.1590/0037-8682-0328-2019

**Published:** 2019-12-20

**Authors:** Eduarda Pena, Sofia Jordão, Maria João Simões, Mónica Oleastro, Isabel Neves

**Affiliations:** 1 Matosinhos Local Health Unit, Infectious Diseases Department, Matosinhos, Porto, Portugal.; 2 National Health Institute Doutor Ricardo Jorge (INSA), Infectious Diseases Department, Lisboa, Portugal.

**Keywords:** Rat-bite fever, Osteomyelitis, Streptobacillus moniliformis

## Abstract

Rat-bite fever is a rarely diagnosed illness caused by *Streptobacillus moniliformis*. Although this disease is distributed worldwide, there have been few cases reported in Europe. Here, we report a case of vertebral osteomyelitis and sternoclavicular septic arthritis caused by *S. moniliformis* in a Portuguese patient previously bitten by a rat. Laboratory diagnosis was performed using molecular identification. This is the first case report of rat-bite fever in Portugal. The case described here serves as a reminder for physicians to consider this diagnosis in patients who have developed fever syndromes after being in contact with rodents.

## INTRODUCTION

We report a case of rat-bite fever (RBF) in a Portuguese woman with no history of recent travel. RBF is a rarely diagnosed illness caused by *Streptobacillus moniliformis* ^1^ ^-^ ^3^. This disease is distributed worldwide and its causative agent is commonly found in the oral flora of rodents. Infection is normally caused by the bite or scratch of a rodent; however, it can also be transmitted through ingestion of contaminated food or water. The incubation period of RBF is generally less than 10 days. Symptoms of RBF include fever, chills, headache, vomiting, migrating arthralgia, myalgia, and rash^4^. The true incidence of this disease is unknown^2^ ^,^ ^3^ and there have been few reported cases throughout Europe^3^ ^-^ ^8^. This case is a reminder for healthcare workers to consider the diagnosis of RBF in patients exhibiting febrile syndromes after coming in contact with a rat.

## CASE REPORT

In August 2016, a 75-year-old woman was admitted to the emergency department of a hospital in the northern region of Portugal with a four-day history of fever, prostration, myalgias, and headache after being bitten by a rat. Her medical history was unremarkable except for hypertension and cervical degenerative disc disease. She was living in a rural area approximately 30 km from Oporto. She denied any recent travel outside of the country. Upon physical examination, she was subfebrile, hypotensive, and displayed incised wounds on two fingers of her left hand. Her neurological exam was normal except for neck stiffness. Laboratory tests revealed a white blood cell count of 14,670/µL (86.3% neutrophils) and an elevated C-reactive protein level of 334 mg/dL. Liver and renal panels were normal. A lumbar puncture detected normal cerebrospinal fluid (CSF) values and a brain computerized tomography (CT) scan was normal. On the first day of hospitalization, the patient was empirically treated with intravenous ceftriaxone (2 g/day). Two blood culture sets were taken upon admission (BD BACTEC Plus Aerobic/F medium). After three days of incubation, both cultures were detected as positive for gram-negative bacteria that were not identified by the phenotypic method (VITEK 2), but later identified as *S. moniliformis* by PCR and Sanger sequencing targeting bacterial 16S rRNA. 

**FIGURE 1:**
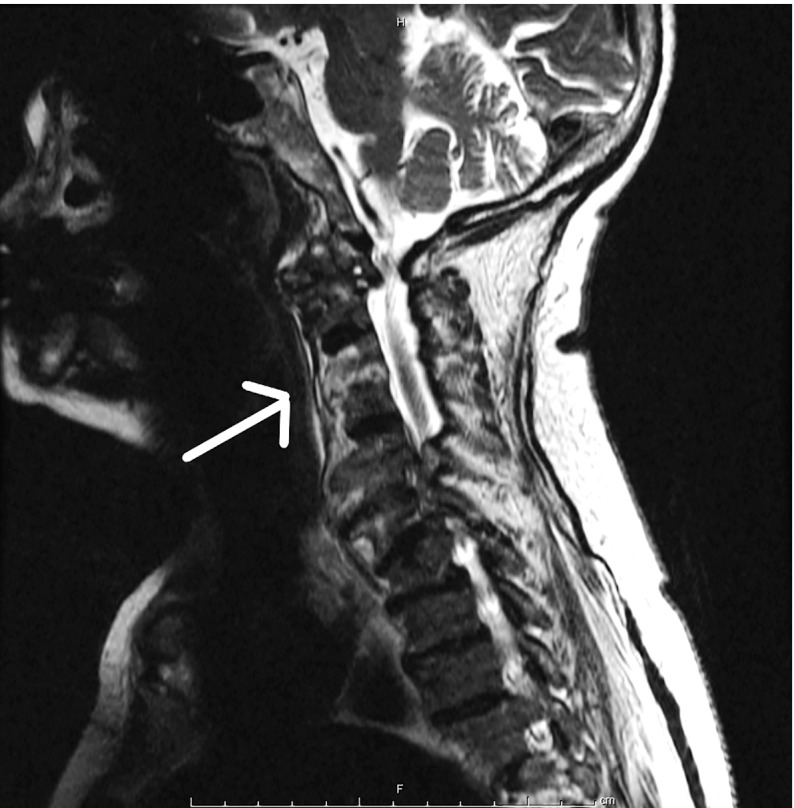
Enhanced T2-weighted magnetic resonance image of the spine of a 75-year-old woman with rat-bite fever. Sagittal view of the cervical spine shows spondylodiscitis and vertebral osteomyelitis from C5 to C7 (indicated by the white arrow).

On the third day after admission, the patient developed worsening neck pain and tetraparesis. Magnetic resonance imaging (MRI) was performed and T2-weighted images showed high signal intensity in the C5, C6, and C7 vertebrae with meningeal enhancement (Figure 1) and the left sternoclavicular joint. These observations provided evidence for a diagnosis of vertebral osteomyelitis and septic arthritis associated with RBF. Orthopedic physicians evaluated the patient and concluded there were no criteria for surgical intervention. Transthoracic echocardiography found no evidence of valvular regurgitation or vegetation. The patient completed 26 days of intravenous ceftriaxone followed by eight months of oral amoxicillin-clavulanate after being discharged from the hospital. During the follow-up period, the patient experienced complete resolution of neck pain and tetraparesis. 

## DISCUSSION

Rat-bite fever is a rarely observed disease in Europe, and there is no accurate data regarding its true incidence because it is not a reportable disease. In the case described here, clinical manifestations were consistent with the literature on acute fever syndrome followed by bone focalization^9^. Associated complications include arthritis, endocarditis, pericarditis, pneumonia, and meningitis. Penicillin is the treatment of choice for RBF, and the duration of intravenous therapy is at least seven days in adult patients^10^. Without treatment, the infection can persist and the mortality rate can be as high as 10%, or even higher (53%) in endocarditis patients^4^. 

Although the patient had a history of recent contact with a rat, the final diagnosis was challenging and achievable only using molecular methods. To our knowledge, this is the fourth reported case of *Streptobacillus*-caused vertebral osteomyelitis^1^ ^,^ ^11^ ^,^ ^12^. The optimal treatment for this type of infection is still uncertain. Our patient completed almost nine months of therapy because imagiological analysis determined that the infection did not resolve within the usual six weeks of therapy. 

The diagnosis of RBF depends on a high level of clinical suspicion based on the adequate questioning about the patient’s recent contact with rodents. Also, it can easily be missed due to both the nonspecific nature of the clinical features and the unusual microbiologic characteristics of *S. moniliformis* (it is an extremely fastidious organism that needs microaerophilic conditions to grow). The latter characteristic makes microbiological diagnosis difficult^2^_,_ especially in common blood culture bottles with sodium polyanethol sulfonate (SPS). Currently, species of *Streptobacillus* can only be reliably distinguished using matrix-assisted laser desorption⁄ionization time-of-flight mass spectrometry (MALDI-TOF MS), Fourier-transform infrared spectroscopy (FTIR), or gene sequence analysis^13^.

In conclusion, following a rat bite, the patient should be placed under clinical surveillance and have their tetanus vaccination history checked. If fever or joint pain arise, physicians should start antibiotic therapy immediately after obtaining blood cultures.
